# Integration of ECG and Point-of-Care Ultrasound in the Diagnosis of Wellens’ Syndrome with Acute Heart Failure: A Case Report

**DOI:** 10.3390/jcm14196982

**Published:** 2025-10-02

**Authors:** Israel Silva, Juan Esteban Aguilar, Andrea Cristina Aragón, Mauricio Sebastian Moreno, Ana Sofia Cepeda-Zaldumbide, Camila Salazar-Santoliva, Jorge Vasconez-Gonzalez, Juan S. Izquierdo-Condoy, Esteban Ortiz-Prado

**Affiliations:** 1 Ministerio de Salud Pública—Centro de Salud, Valencia, Los Rios, 120610 Ecuador; 2 Ministerio de Salud Pública—Centro de Salud, San Antonio de Pichincha, Quito, 170102 Ecuador; 3 Ministerio de Salud Pública—Centro de Salud, Lasso, Cotopaxi, 050102 Ecuador; 4 One Health Research Group, Universidad de las Americas, Quito 170137, Ecuador

**Keywords:** Wellens pattern, acute heart failure, electrocardiography, T-wave inversion

## Abstract

**Introduction:** Twelve-lead electrocardiography (ECG) remains an essential diagnostic tool for patients presenting with chest pain. Timely recognition of specific electrocardiographic patterns is critical for guiding reperfusion strategies and predicting adverse outcomes. Among these, Wellens’ pattern is a high-risk marker of critical left anterior descending (LAD) artery stenosis and an impending anterior myocardial infarction. Although typically described in clinically stable patients without heart failure, its occurrence in the setting of acute decompensation is rare. **Case Report:** We report the case of a 66-year-old male with hypertension, obesity, and active smoking who presented with exertional chest pain, dyspnea, and signs of acute heart failure. Initial ECG revealed biphasic T waves in V2–V4, consistent with type A Wellens’ pattern. Laboratory evaluation demonstrated elevated troponin I, while point-of-care ultrasound (POCUS) identified systolic and diastolic dysfunction, lateral wall hypokinesia, pericardial effusion, and cardiogenic pulmonary edema. The patient received acute management with antiplatelet therapy, statins, diuretics, and anticoagulation, followed by referral for coronary angiography. This revealed critical stenosis (>90%) of the proximal LAD, successfully treated with percutaneous coronary intervention and drug-eluting stent implantation. The in-hospital course was uneventful, and guideline-directed medical therapy was optimized at discharge, including dual antiplatelet therapy, beta-blocker, renin–angiotensin system inhibitor, and SGLT2 inhibitor. **Conclusions:** This case highlights the need for early recognition of Wellens’ pattern, even in atypical contexts such as acute heart failure. Integrating ECG interpretation with bedside POCUS facilitated diagnostic accuracy and guided an early invasive strategy, preventing extensive myocardial infarction. In resource-limited settings, strengthening frontline diagnostic capabilities and referral networks is crucial to improving patient outcomes.

## 1. Introduction

Twelve-lead electrocardiography is an essential diagnostic tool for patients presenting with chest pain, as the timely recognition of specific electrocardiographic patterns is crucial for guiding diagnosis. It enables clinicians to distinguish ischemic etiologies, expedite therapeutic decision-making—such as directing emergent reperfusion strategies—and predict adverse outcomes [1,2]. However, clinical assessment becomes more challenging in the context of non–ST-segment elevation acute coronary syndromes (NSTE-ACS), where electrocardiographic findings may be subtle or nonspecific [3].

A representative example is the Wellens pattern, also known as the T-wave inversion syndrome of the left anterior descending artery [4]. Two morphologies have been described: type A, observed in approximately 24% of patients, characterized by biphasic T waves in leads V2–V3; and type B, present in about 75% of cases, characterized by deep and symmetric T-wave inversion in V2–V3, sometimes extending to V6 [4,5,6]. This pattern is strongly associated with critical stenosis of the left anterior descending (LAD) artery [5]. Symmetrically inverted or biphasic T waves in the anterior precordial leads are widely recognized as markers of an impending LAD-territory infarction. In this regard, Kobayashi et al. reported a sensitivity of 24.6% and a specificity of 96.2% for proximal LAD occlusion when this finding is present [7]. This syndrome represents a pre-infarction state; therefore, it is important to emphasize that delayed recognition or misguided treatment could result in a large anterior wall myocardial infarction [8]. Such an event may lead to complications including anterior wall hypokinesia, necrosis, or rupture, ultimately culminating in heart failure.

For over a decade, Wellens and related patterns have been classified as electrocardiographic equivalents of ST-elevation myocardial infarction (STEMI), as they indicate an acute epicardial occlusion of a major coronary artery [9]. Early recognition is critical, since failure to identify these patterns may result in significant myocardial injury and adverse clinical outcomes. Moreover, the association of Wellens syndrome with acute heart failure is rare. Most reported cases describe the absence of symptoms or signs of acute heart failure, or make no mention of it [10,11,12,13,14]. Only two reports have documented the presence of acute heart failure. Okobi et al. described a case of Wellens syndrome with deep T waves in leads V2–V3, associated with COVID-19 pneumonia, pleural effusions, and congestive heart failure [4]. Similarly, Zhou et al. reported that among 138 patients with Wellens syndrome, one presented with heart failure [15]. Here, we present a case of Wellens syndrome manifesting in the atypical context of acute heart failure, highlighting its diagnostic challenges and therapeutic implications in a resource-limited setting.

## 2. Methodology

This case report was prepared in accordance with the CARE guidelines for case reports to ensure accuracy, transparency, and clinical relevance [16]. Prior to drafting, informed consent was obtained from the patient. The patient was informed that only clinical information would be used in the report and that no identifying data—such as name, home address, phone number, email, or identification number—would be included. Furthermore, it was explained that any images used would be strictly medical in nature, such as electrocardiograms or imaging studies, and that no photographs permitting patient identification would be employed at any time.

## 3. Case Presentation

A 66-year-old Caucasian male with a history of arterial hypertension diagnosed approximately 12 years earlier, without established pharmacologic treatment, presented to the emergency department. Additional risk factors included class I obesity, sedentary lifestyle, and active tobacco use of about one pack per day. He denied any surgical history, drug allergies, or regular medication use. His family history was notable for sudden cardiac death in his father at the age of 80.

The patient reported a 72-h history of mild precordial chest discomfort triggered by physical exertion, particularly when walking short distances, which improved with rest. He also described exertional dyspnea with mild functional limitation, consistent with New York Heart Association (NYHA) class II (Table 1).

Prior to arrival, he experienced a 30-min episode of retrosternal chest pain of oppressive quality, rated 8/10 in intensity, precipitated by exertion. This was accompanied by sudden-onset moderate dyspnea, nausea without vomiting, and profuse diaphoresis.

On physical examination, the patient was alert, oriented, and afebrile but appeared in visible distress with marked orthopnea. Vital signs included blood pressure of 160/85 mmHg, heart rate of 80 bpm, respiratory rate of 24 breaths per minute, and oxygen saturation of 85% on room air. The skin was warm with preserved capillary refill. Significant bilateral lower extremity edema (+++) and grade II jugular venous distension were noted. Cardiac auscultation revealed a regular rhythm without murmurs, gallops, or extra heart sounds. Peripheral pulses were present and symmetrical. Pulmonary examination demonstrated bilateral basal crackles, consistent with Killip class II. The remainder of the examination was unremarkable.

### 3.1. Diagnostic Approach

**Figure 1:**
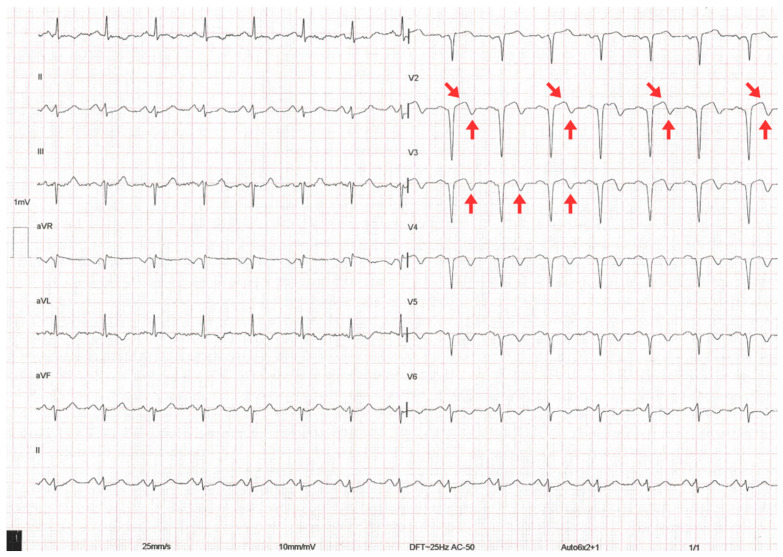
The 12-lead electrocardiogram on admission showing biphasic T waves (red arrows), consistent with Wellens type A pattern.

A 12-lead ECG performed within the first 10 min of arrival showed sinus rhythm at 88 bpm, PR interval of 160 ms, QRS duration of 110 ms, and corrected QT interval (QTc) of 440 ms. Biphasic T waves were present in the anteroseptal leads (V2–V4), consistent with a type A Wellens pattern (Figure 1). However, other diagnoses to consider with these electrocardiographic findings include central nervous system injury (cerebral T waves), right bundle branch block, pulmonary embolism, hypertrophic cardiomyopathy, and left ventricular hypertrophy, the latter suggested in this case by a Peguero–Lo Presti index >2.8 mV.

Initial laboratory evaluation showed a normal complete blood count, serum creatinine of 0.63 mg/dL (eGFR 102.69 mL/min/1.73 m^2^ by CKD-EPI), and blood urea nitrogen of 44 mg/dL. Troponin I was elevated at 0.16 ng/mL (reference < 0.03 ng/mL), indicating acute myocardial injury (Table 2).

**Figure 2:**
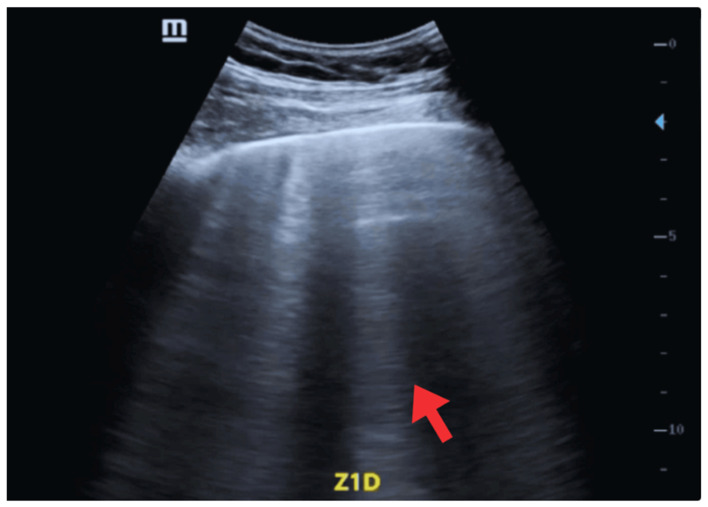
POCUS—Oblique window, Zone 1 (Right lung). Findings compatible with cardiogenic alveolar-interstitial syndrome (red arrow).

For POCUS, two transducers were used: a 2–5 MHz cardiology sector probe for cardiac evaluation (depth 10–15 cm) and a 7–12 MHz linear probe for pleural and pulmonary evaluation (depth 5–6 cm). The pulmonary evaluation included eight areas (anterior-superior, anterior-inferior, lateral-superior, and lateral-inferior of each hemithorax) based on the standardized protocol proposed by Volpicelli et al. [17]. B-lines were quantified in every intercostal space, defined as vertical hyperechoic artifacts arising from the pleura, extending to the lower edge of the screen, obliterating A-lines, and moving synchronously with pleural sliding [18]. A pathological finding was defined as ≥3 B-lines per intercostal space. Lung ultrasound revealed a regular pleural line with findings consistent with cardiogenic alveolar-interstitial syndrome, including B-lines covering 50–60% of the lung fields (Figure 2).

**Figure 3:**
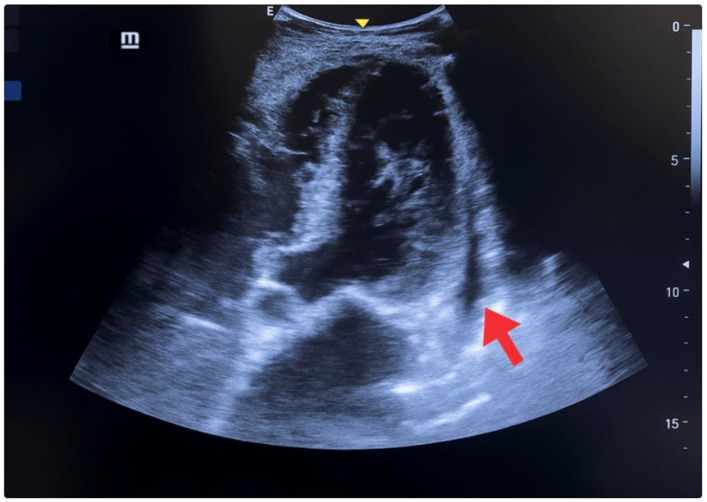
POCUS—Apical 5 chambers view. Small, non-circumferential pericardial effusion, systolic and diastolic dysfunction (red arrow), lateral wall hypokinesia, interventricular septum and posterior wall thickening, and chamber enlargement.

Cardiac evaluation was performed using parasternal and apical windows. Left ventricular function was assessed both qualitatively (“eyeballing”) and quantitatively. Using the Simpson method (apical two- and four-chamber views), the ejection fraction was estimated at 40% (normal > 55%), consistent with moderate systolic dysfunction. The E-point septal separation (EPSS) measured on the parasternal long axis was 10 mm, correlating with an ejection fraction <50%. The left ventricular shortening fraction was 18% (normal 25–45%). Additional findings included lateral wall hypokinesia, eccentric remodeling with cavity enlargement (indexed end-diastolic volume >76 mL/m^2^), and interventricular septal thickening. A small, non-circumferential pericardial effusion was also observed (Figure 3) [19,20,21].

An important diagnostic challenge was interpreting the elevated troponin level. While troponin is highly sensitive for myocardial injury, it lacks specificity, as elevations may occur in ischemia, myocarditis, or decompensated heart failure [22,23]. In this case, interpretation was integrated with the clinical context, ECG, and echocardiographic findings to ensure diagnostic precision, guide therapy, and optimize prognosis.

### 3.2. Therapeutic Intervention

On admission, the patient received intravenous nitroglycerin to relieve chest pain and reduce preload and afterload, supplemental oxygen at 4 L/min to maintain SpO_2_ >94%, aspirin 300 mg orally, clopidogrel 300 mg orally, and rosuvastatin 40 mg orally.

Based on echocardiographic findings and clinical signs of congestion, a diagnosis of acute heart failure secondary to ischemic heart disease was established. Given the absence of prior history or treatment for heart failure, this presentation was considered a first episode of acute decompensated heart failure, corresponding to hemodynamic profile B. Intravenous furosemide 40 mg was administered as decongestive therapy.

During 36 h of observation in a primary care unit, his pharmacologic regimen was optimized: aspirin 100 mg once daily, clopidogrel 75 mg once daily, rosuvastatin 40 mg once daily, and furosemide 40 mg IV every 8 h, with favorable fluid balance. He was subsequently referred to a higher-level facility for coronary angiography, at which time enoxaparin 80 mg subcutaneously every 12 h was added.

**Figure 4:**
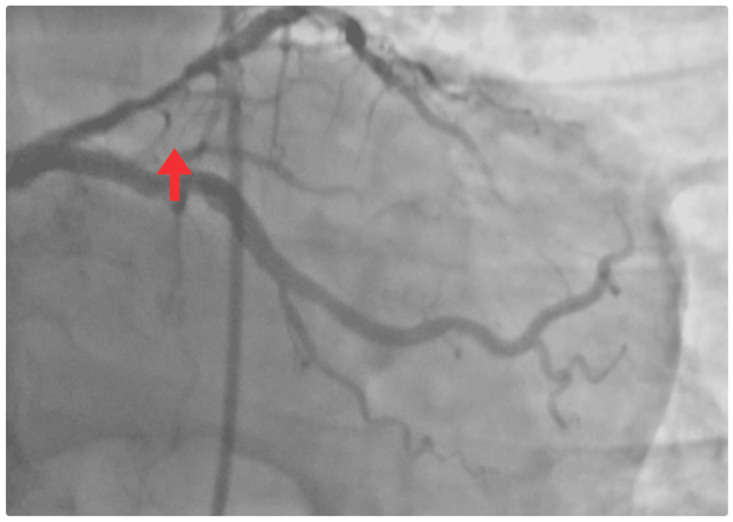
Coronary angiogram. Occlusive lesion in the proximal LAD segment (red arrow).

Coronary angiography revealed no significant left main stenosis. However, a severe proximal LAD lesion with >90% luminal stenosis was identified. Chronic atherosclerotic disease was also observed in the circumflex artery without hemodynamic significance, while other vessels were free of relevant disease (Figure 4).

**Figure 5:**
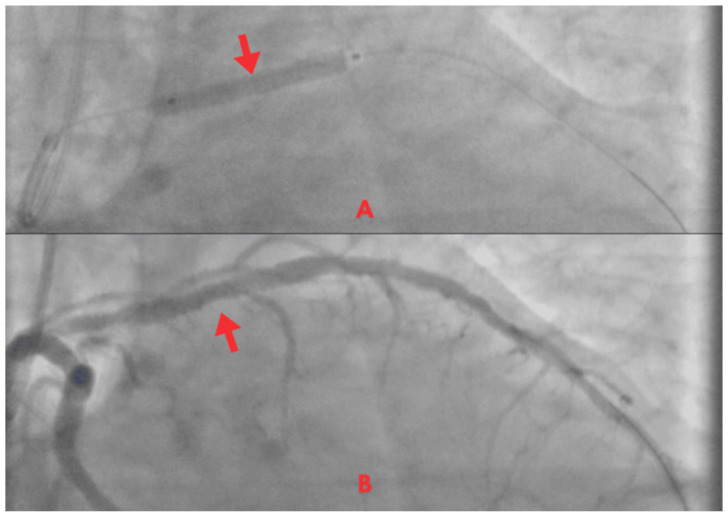
Coronary angiogram. (**A**) PCI with stent implantation in the proximal LAD (red arrow). (**B**) Post-procedure with restored TIMI 3 coronary flow (red arrow).

Percutaneous coronary intervention (PCI) was indicated. Via radial access, angioplasty with implantation of a drug-eluting stent (DES) was successfully performed in the proximal LAD (Figure 5A), restoring TIMI grade 3 flow and achieving a satisfactory angiographic outcome (Figure 5B). The procedure was uneventful.

### 3.3. Clinical Follow-Up and Outcomes

Following successful revascularization, the patient was transferred to a step-down unit. The in-hospital course was uneventful, with hemodynamic stability, resolution of congestion, and no recurrence of angina.

At discharge, guideline-directed medical therapy was initiated: aspirin 100 mg and clopidogrel 75 mg daily, rosuvastatin 40 mg daily, carvedilol 6.25 mg every 12 h, enalapril 5 mg daily, furosemide 40 mg every 12 h, and dapagliflozin 10 mg daily. A structured plan for dose titration was recommended.

Non-pharmacologic measures included smoking cessation counseling, a sodium-restricted diet, lifestyle modifications, and weight reduction strategies. Follow-up with cardiology was scheduled within 7–10 days, with serial echocardiography at 48 h and again at 4–6 weeks, alongside laboratory monitoring. The aim was optimal symptom control, prevention of recurrent cardiovascular events, and improvement of long-term outcomes.

**Figure 6:**
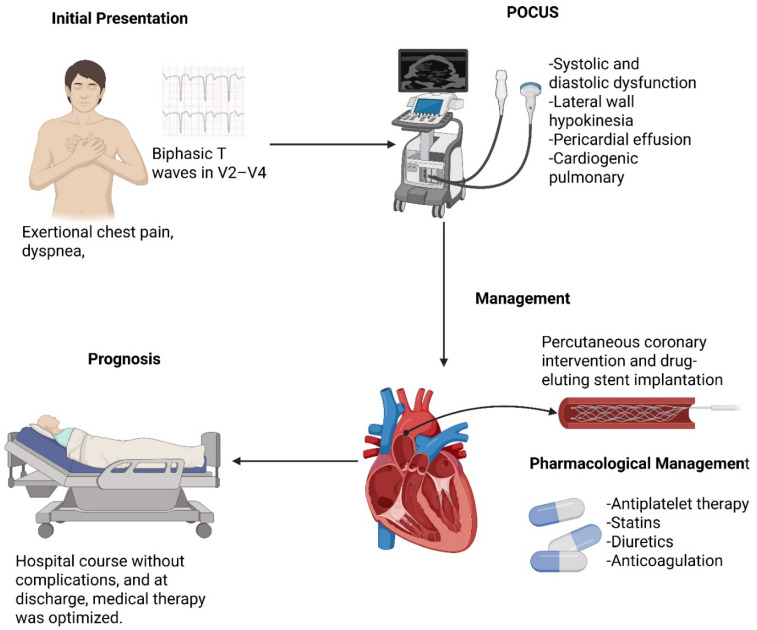
Patient Clinical Course Summary.

At 7 days post-PCI, the patient was in good general condition, hemodynamically stable, and free of chest pain (Figure 6). Previous ischemic changes (biphasic T waves) had been resolved, with no new abnormalities. No signs of heart failure were observed on examination. The patient reported good adherence to prescribed therapy, and counseling regarding cardiovascular risks was reinforced. As no evidence of congestion was present, furosemide was withdrawn.

At the 1-month follow-up, the patient remained asymptomatic, with good tolerance of all medications and no reported adverse drug effects. A new electrocardiogram showed no signs of active ischemia. Left ventricular ejection fraction, reassessed by echocardiography, was preserved. No hospital re-admissions or recurrent cardiovascular events occurred during this period. However, LDL-cholesterol levels remained above target (104 mg/dL), consistent with very high cardiovascular risk. Accordingly, rosuvastatin 40 mg daily was switched to atorvastatin 80 mg daily plus ezetimibe 10 mg daily. All other medications were continued, and enrollment in a cardiac rehabilitation program was recommended, along with monthly follow-up in the cardiology outpatient clinic.

## 4. Discussion

This case underscores the importance of recognizing Wellens’ pattern in a resource-limited setting and in an atypical clinical presentation such as acute heart failure. Although this electrocardiographic pattern has traditionally been described in clinically stable and asymptomatic patients at the time of ECG acquisition, in the present case it appeared in the context of significant hemodynamic congestion—posing a diagnostic challenge, particularly when elevated cardiac biomarkers are overemphasized without clear clinical correlation [6]. It was therefore important to rule out other causes of troponin elevation such as inflammatory heart disease, pulmonary embolism, renal failure, or sepsis [24].

Wellens’ pattern should be regarded as a cardiologic emergency that requires early invasive coronary assessment. Timely intervention can prevent major adverse cardiac events, including extensive anterior myocardial infarction. The condition is associated with several risk factors, including hypertension, which promotes arterial wall thickening, atherosclerotic plaque development, and increased vulnerability to rupture; diabetes, characterized by hyperglycemia, insulin resistance, dyslipidemia, endothelial dysfunction, vasoconstrictive tendency, and a prothrombotic state; a sedentary lifestyle, which contributes to obesity and chronic inflammation, thereby promoting atherosclerosis; and smoking, which accelerates the progression of atherosclerosis in the abdominal aorta as well as the iliac and femoral arteries [25,26,27,28]. In the present case, the patient had hypertension, class I obesity, and active smoking as predisposing factors [8]. The literature emphasizes the importance of maintaining high clinical suspicion even in the absence of active chest pain or troponin elevation, as this pattern reflects a transient ischemic phase with a high risk of imminent complete coronary occlusion [8,29]. Consequently, Wellens’ pattern represents a high-risk electrocardiographic marker within the acute coronary syndrome spectrum, with significant diagnostic, therapeutic, and prognostic implications [30,31,32].

Although many cases of Wellens’ syndrome have been described in the literature, few have presented with heart failure. The syndrome is typically associated with asymptomatic patients. However, it is noteworthy that Wellens’ original descriptions were based on cases of unstable angina, which could be explained by the absence of modern biomarkers such as high-sensitivity troponin at that time [33]. Angiographic and clinical characteristics of unstable angina patients with ECG evidence of critical proximal LAD narrowing further support that, under current definitions, these patterns may correspond to myocardial infarction [33].

In 2023, three cases were reported in which Wellens’ pattern progressed from type A to type B within an average of 12 h [5]. This highlights the priority of early recognition to prevent progression, complications, or even death. Recognition of Wellens’ pattern in patients presenting with acute heart failure should therefore prompt immediate invasive evaluation [34]. In this case, the patient was initially managed in a primary care facility without interventional capacity. This underscores both the structural limitations of health systems in resource-constrained environments and the variability in the clinical presentation of Wellens’ syndrome. Maintaining a high index of suspicion—even in complex or atypical scenarios—is essential, as timely ECG interpretation may be decisive for patient outcomes.

Beyond electrocardiography, bedside POCUS can further shorten time-to-diagnosis and guide triage in resource-limited settings. In patients with suspected ACS and dyspnea, focused cardiac and lung ultrasound rapidly identifies left-ventricular systolic dysfunction, regional wall-motion abnormalities, pericardial effusion, and cardiogenic pulmonary edema (B-lines), refining the assessment of ischemia-driven decompensation and supporting early, targeted therapy. In the present case, POCUS findings (lateral wall hypokinesia, moderate left ventricular dysfunction, small pericardial effusion, and diffuse B-lines) complemented ECG interpretation and reinforced the decision to pursue early invasive angiography. Evidence from low- and middle-income countries suggests that POCUS improves diagnostic accuracy, reduces unnecessary testing, and expedites clinical disposition when advanced imaging or interventional cardiology are not immediately available [35]. Moreover, our group has shown in a high-risk endocrine emergency that POCUS can be decisive for narrowing a broad differential and accelerating definitive management under hemodynamic instability, underscoring its value as an adaptable first-line imaging tool in constrained environments [36].

Acute heart failure itself is associated with a substantial increase in mortality. The French FAST-MI registry demonstrated that patients with congestive heart failure had a significantly higher risk of death, with an adjusted hazard ratio of 1.55. Furthermore, among this subgroup, those not treated with renin–angiotensin–aldosterone system (RAAS) inhibitors or beta-blockers exhibited higher mortality compared to those appropriately treated [37].

According to the 2023 ESC Guidelines for the management of acute coronary syndromes and the 2021 ACC/AHA/SCAI Guideline for Coronary Revascularization, patients with Wellens’ pattern should be managed as high-risk NSTE-ACS, for which an early invasive strategy (within 24 h, or sooner if unstable) is strongly recommended. Guideline-directed medical therapy includes dual antiplatelet therapy (aspirin plus a P2Y12 inhibitor), high-intensity statin, beta-blocker, and renin–angiotensin system inhibition, unless contraindicated. In this case, our patient was treated in accordance with these recommendations, with immediate initiation of aspirin, clopidogrel, and rosuvastatin, followed by anticoagulation and subsequent PCI [38,39].

It is important to highlight that, unlike in STEMI, fibrinolysis is not indicated for Wellens’ syndrome. The underlying pathophysiology is a critical fixed stenosis of the LAD rather than an acute thrombotic occlusion, which explains the poor outcomes of fibrinolysis in this context [40,41]. However, in resource-constrained settings where primary PCI is not immediately available, some institutions may consider fibrinolysis in cases of evolving STEMI during observation, while arranging transfer to a PCI-capable center (pharmaco-invasive strategy) [42]. In our patient, fibrinolysis was not necessary as urgent PCI was performed, consistent with current recommendations.

In many Latin American countries, however, limited access to PCI necessitates fibrinolysis as an alternative strategy [42]. Despite its utility in other contexts, fibrinolysis has little role in Wellens’ syndrome. Patients with this condition generally respond poorly to medical therapy alone, as the underlying pathology is not an acute thrombotic occlusion but rather a critical fixed stenosis. Such lesions are more effectively treated with mechanical revascularization, which remains the definitive therapeutic approach [8]. With conservative management, up to 75% of these patients progress to anterior myocardial infarction within 1–23 days, underscoring the need for urgent revascularization [43]. This further emphasizes the importance of strengthening diagnostic capabilities at the primary care level and ensuring the functionality of streamlined referral networks.

Delays in PCI after acute myocardial infarction are associated with a significant increase in cardiovascular complications. In STEMI, early revascularization (<12 h from symptom onset or <90 min from first medical contact) reduces mortality, limits infarct size, and prevents mechanical complications and arrhythmias. Conversely, delaying the procedure beyond 36 h, particularly in patients with persistent ischemia or hemodynamic instability, increases the likelihood of post-infarction heart failure, adverse ventricular remodeling, and higher short- and medium-term mortality [44,45]. These findings highlight the importance of prioritizing early revascularization to minimize myocardial damage and improve outcomes. In this case, delaying coronary angioplasty could have worsened the acute heart failure presentation [45].

Early recognition and timely implementation of an invasive strategy are therefore critical to preventing myocardial injury [46]. In NSTE-ACS, current recommendations stress individualized risk stratification at presentation, after which an immediate, early, or delayed invasive strategy should be pursued depending on the classification [47]. Patients at very high risk should undergo urgent coronary angiography within 2 h of admission, whereas those with at least one intermediate-risk criterion should receive angiography within 72 h. In contrast, low-risk patients may be managed conservatively [47].

Finally, this case also illustrates the importance of secondary prevention. Given the history of acute coronary syndrome, the patient is classified as very high cardiovascular risk, with a therapeutic LDL-cholesterol target <55 mg/dL. At the one-month follow-up, LDL levels remained above this threshold despite high-intensity statin therapy. Consequently, treatment was optimized by switching the statin regimen and adding ezetimibe, in accordance with the most recent dyslipidemia management guidelines [48].

It is important to acknowledge the limitations of this case report. As it involves a single patient and lacks a control group, establishing causality is not possible. Furthermore, the findings cannot be generalized to other populations. These limitations should be considered when interpreting the results and highlight the need for additional reports to improve understanding of the condition described.

## 5. Conclusions

Wellens’ pattern should be recognized as a high-risk electrocardiographic warning sign within the acute coronary syndrome spectrum—even in atypical scenarios such as acute heart failure. In this case, timely ECG interpretation, complemented by focused bedside POCUS to characterize ventricular dysfunction, pericardial effusion, and cardiogenic pulmonary edema, supported an early invasive strategy that prevented progression to extensive anterior myocardial infarction and enabled favorable short-term outcomes. Beyond individual management, this case underscores system-level priorities in resource-limited settings: strengthening frontline diagnostic capabilities (ECG and POCUS proficiency), ensuring rapid referral pathways to interventional cardiology, and implementing guideline-directed medical therapy after revascularization. Prioritizing these elements can shorten time-to-diagnosis, reduce avoidable myocardial injury, and improve patient-centered outcomes in low- and middle-income environments.

## Figures and Tables

**Table 1:** Clinical and Therapeutic Summary of Patient Evolution from Admission to Discharge.

Date	Symptoms and Signs	Paraclinical and Laboratory Results	Imaging Results	Treatment
2 May	Intermittent precordial pain with exertion, mild intensity, lasting 72 h, accompanied by dyspnea on minor exertion (NYHA II)	-	-	-
5 May	Acute episode of oppressive retrosternal chest pain (30 min, 8/10), with sudden moderate dyspnea, nausea, and profuse diaphoresis. He presented to the primary emergency department. Initial evaluation: BP 160/85 mmHg, SatO_2_ 85%, signs of pulmonary and peripheral congestion.	-ECG: Biphasic T waves were present in the anteroseptal leads (V2–V4), consistent with a type A Wellens pattern. -Laboratory: elevated troponin I (0.16 ng/mL), (reference < 0.03 ng/mL).	POCUS: Ejection fraction of 40% (moderate systolic dysfunction), shortening fraction of 18%, EPSS of 10 mm, lateral wall hypokinesia, eccentric remodeling with ventricular dilatation, thickened interventricular septum, and a small non-circumferential pericardial effusion. At the lung level, diffuse B lines (50–60% of lung fields) were present, a pattern consistent with alveolar-interstitial syndrome of cardiogenic origin.	Initial treatment: oxygen, IV nitroglycerin, ASA 300 mg PO, clopidogrel 300 mg PO, rosuvastatin 40 mg PO, IV furosemide.
6–7 May	Mild retrosternal chest pain, absence of dyspnea, and improved ventilatory mechanics.	ECG: Biphasic T waves were present in the anteroseptal leads (V2–V4), consistent with a type A Wellens pattern.	POCUS: Decreased pulmonary B lines in both lung fields (40–50%).	Referral to a more complex center. Enoxaparin 80 mg SC every 12 h is added.
8 May	Mild retrosternal chest pain, absence of dyspnea, and improved ventilatory mechanics.	-	-	Coronary angiography: critical proximal LAD stenosis (>90%). Chronic atherosclerotic disease in the circumflex artery, without significant obstructive lesions. Percutaneous coronary intervention (PCI): angioplasty + drug-eluting stenting in the proximal LAD (TIMI 3 flow).
9–11 May	Hospital stay in an intermediate care unit.	ECG: -Rhythm: sinus (positive P wave in L1, L2, and aVF; each P wave is followed by a QRS complex). -Heart rate: 80 beats per minute. -Electrical axis: between (−60° left axis deviation). -P wave: duration ≤ 120 ms, amplitude ≤ 2.5 mm in limb leads. PR interval: 160 ms. -QRS complex: duration ≤ 110 ms. -ST segment: isoelectric. -T wave: positive in most leads, except aVR and V1. -Corrected QT interval (QTc): 400 ms. -Findings of left ventricular hypertrophy.	POCUS: -Absence of pulmonary and cardiac congestion. -Regular pleura, without thickening. -Marked reduction in B lines (<20%) in affected lung fields. -Reappearance of predominant A lines. -Absence of consolidation. Findings compatible with resolution of cardiogenic alveolar-interstitial syndrome after depleting therapy. -Ejection fraction (Simpson): 50–55% -EPSS: 7 mm (previously 10 mm). -Shortening fraction: 25%. -Less evident lateral hypokinesia. -Left chambers with reduced diameter and indexed end-diastolic volume (≤70 mL/m^2^). -Mild, stable, non-progressive pericardial effusion.	-
12 May	Favorable outcome: hemodynamic stability, no recurrence of angina, resolution of congestion. Hospital discharge	Normal ECG and laboratory.	-	Hospital discharge -Double antiplatelet therapy (ASA 100 mg/d + clopidogrel 75 mg/d). -Rosuvastatin 40 mg/d. -Carvedilol 6.25 mg every 12 h. -Enalapril 5 mg/d. -Furosemide 40 mg every 12 h. -Dapagliflozin 10 mg/d. -Lifestyle recommendations and outpatient follow-up.
19 May	Outpatient cardiology follow-up consultation (7 days after discharge). Clinical reevaluation and therapeutic adjustment.	-	-	-

**Table 2:** Laboratory Test Results.

Test	Result	Reference Range
**Complete blood count**		
White Blood Cells (WBC)	9.8 × 10^9^/L	4.0–10.0 × 10^9^/L
Red Blood Cells (RBC)	5.3 × 10^12^/L	4.5–6.0 × 10^12^/L
Hemoglobin (Hb)	16.1 g/dL	13.5–17.5 g/dL
Hematocrit (Hct)	47%	41–53%
Mean Corpuscular Volume (MCV)	89 fL	80–96 fL
Mean Corpuscular Hemoglobin (MCH)	30 pg	27–33 pg
Mean Corpuscular Hemoglobin Concentration (MCHC)	34 g/dL	32–36 g/dL
Platelets	230 × 10^9^/L	150–400 × 10^9^/L
**Renal Function**		
Blood Urea Nitrogen (BUN)	44 mg/dL	10–45 mg/dL
Creatinine	0.63 mg/dL	0.6–1.3 mg/dL
Estimated glomerular filtration rate	102.69 mL/min/1.73 m^2^	>90 mL/min/1.73 m^2^
Cardiac profile		
Troponin I	0.16 ng/mL	<0.03 ng/mL
**Arterial Blood Gas (ABG)**		
pH	7.41	7.35–7.45
pCO_2_	44 mmHg	35–45 mmHg
pO_2_	84 mmHg	80–100 mmHg
HCO_3_^−^	22 mmol/L	22–26 mmol/L
Base Excess (BE)	−3 mmol/L	−2–+2 mmol/L
O_2_ Saturation (SaO_2_)	91%	95–100%
Sodium	139	136–145 mmol/L
Potassium	3.7	3.5–5.1 mmol/L
Chloride	104	98–107 mmol/L

## Data Availability

The original contributions presented in this study are included in the article. Further inquiries can be directed to the corresponding author.

## Abbreviations

The following abbreviations are used in this manuscript:

POCUS	point-of-care ultrasound
LAD	left anterior descending
STEMI	ST-elevation myocardial infarction
NYHA	New York Heart Association
ECG	electrocardiogram
LVH	left ventricular hypertrophy
DES	drug-eluting stent
